# Hepatitis A virus subgenotyping based on RT-qPCR assays

**DOI:** 10.1186/s12866-014-0296-1

**Published:** 2014-11-25

**Authors:** Coralie Coudray-Meunier, Audrey Fraisse, Camélia Mokhtari, Sandra Martin-Latil, Anne-Marie Roque-Afonso, Sylvie Perelle

**Affiliations:** Université Paris-Est, ANSES, Food Safety Laboratory, Enteric viruses unit, 23 Avenue du Général de Gaulle, 94706 Maisons-Alfort, cedex, France; AP-HP, Hôpital Paul Brousse, Virologie, Villejuif, 94804 France; Univ Paris-Sud, UMR-S 785, Villejuif, 94804 France; INSERM U785, Villejuif, 94804 France

**Keywords:** Hepatitis A virus, RT-qPCR assays, Genotyping

## Abstract

**Background:**

The hepatitis A virus (HAV) is the most frequent cause of viral hepatitis worldwide and is recognized as one of the most widespread foodborne pathogens. HAV genotypes and subtypes differ in their geographic distribution and the incidence of HAV infection varies considerably among countries, and is particularly high in areas with poor sanitation and hygiene. Phylogenetic analyses are traditionally used in clinical microbiology for tracing the geographic origin of HAV strains. In food microbiology, this approach is complicated by the low contamination levels of food samples. To date, real-time reverse-transcription PCR has been one of the most promising detection methods due to its sensitivity, specificity and ability to deliver quantitative data in food samples, but it does not provide HAV subtyping information.

**Results:**

Six subtype-specific RT-qPCR assays were developed for human HAV. The limit of detection of HAV was 50 genome copies/assay for subtype IIB, 500 genome copies assay for IA, IB, IIA and IIIB and 5000 genome copies/assay for IIIA. The specificity of the assays was evaluated by testing reference isolates and *in vitro* HAV RNA transcripts. No significant cross reactivity was observed. Subtyping results concordant with sequencing analysis were obtained from 34/35 clinical samples. Co-infection with a minor strain of a different subtype was suggested in 5 cases and a recombinant event in one case.

**Conclusions:**

These RT-qPCR assays may be particularly useful for accurately tracing HAV in low-level contaminated samples such as food matrices but also to allow co-infection identification in human samples.

## Background

Hepatitis A virus (HAV) is a small, non-enveloped hepatotropic virus classified into the *Hepatovirus* genus within the *Picornaviridae* family. Its genome consists of an approximately 7.5 kilobase positive single-strand RNA comprising a 5’ untranslated region (5’UTR), a single open reading frame (ORF) that encodes both structural and non-structural proteins, and a 3’ UTR with a short poly(A) tail. There is only one serotype of HAV. Genomic characterization of HAV has been carried out mainly by sequencing of strains from different geographic regions of the world. Firstly, using a short fragment of the VP1/2A junction region, strains were classified in to seven genotypes on the basis of >15% nucleotide variation between isolates, and the subgenotypes with >7.5% to <15% nucleotide variation [1]. Then, the complete genomic data indicated that genotypes II and VII should be considered a single genotype, based upon the complete VP1 sequence [2; 3]. So, by sequencing of the VP1/2A junction and the VP1 gene, three genotypes (I, II, III) divided in two subtypes (A and B) have been described for humans and three others (IV, V, VI) for primates [1-3].

HAV infection is the leading worldwide cause of acute viral hepatitis [4,5]. There are an annual estimated of 1.5 million cases of hepatitis A worldwide [6]. Optimal use of vaccination can significantly reduce the hepatitis A disease burden and the World Health Organization position on hepatitis A vaccines depend on the level of endemicity in countries. In highly endemic countries, large-scale vaccination programmes are not recommended. In countries of intermediate endemicity, large-scale childhood vaccination may be considered as a supplement to health education and improved sanitation. And in regions of low endemicity, vaccination against hepatitis A is indicated for individuals with increased risk of contracting the infection such as travelers to areas of intermediate or high endemicity [7]. HAV’s geographical distribution is dependent on socioeconomic development and sanitation levels. In areas with high and very high endemicity (Africa, Middle East, India, Central and South America), where infections are mostly asymptomatic and epidemics are rare, 50% seroprevalence is reached between the ages of 5 and 14 [8]. In areas with moderate endemicity (Eastern Europe and south-eastern Asia), 50% seroprevalence is reached between the ages of 14 and 34 and epidemics can occur within the general population. In areas with low endemicity (North America, Western Europe and Australia), most of the population is still susceptible to HAV, particularly people over 50 years old, and the risk of fulminant hepatitis is higher.

HAV is transmitted mainly by the fecal-oral route, either by person-to-person contact or by ingestion of contaminated water and food, particularly shellfish, soft fruits and raw vegetables [9-16]. HAV is stable in the environment and is particularly resistant to disinfectants, heating, pressure and low pH [4,17]. Contamination may occur during growth in the field as well as during processing, storage, distribution or final preparation. In developed countries, low incidence and low vaccine coverage have led to a high proportion of susceptible individuals, which creates a potential for expanded hepatitis A outbreaks when contaminated products are widely distributed [8].

The development of sensitive, reliable techniques for the detection of HAV in food and water samples contributes to the safety of these products [18]. However, detection of HAV on the basis of its infectivity is complicated by the absence of a reliable cell culture method and the low contamination levels of food samples. HAV detection is currently based on nucleic acid testing methods. The International Organization for Standardization/Technical specification (ISO/TS) 15216 standard was published in the first half of 2013 and will be published as ISO standard methods after validation. These protocols target the 5’UTR which shows the lowest diversity across HAV genotypes [19-22]. Currently, HAV genotyping relies on amplification, sequencing and phylogenetic analysis of a portion of the viral genome. However, these techniques are time-consuming and may lack sensitivity, particularly with food samples, where the level of contamination by enteric viruses is often very low. Alternative approaches for HAV genotyping in complex samples (food, environmental) may help to better manage the risk. Indeed, although genotypes I and III are the most frequently reported worldwide, HAV genotypes and HAV strains differ in their geographic distribution [23,24]; strain genotyping can thus give clues to understanding food contamination routes. Currently, very few studies describe alternatives to sequencing for HAV genotyping. In recent years single-nucleotide polymorphism (SNP) genotyping has become an area of intense investigation and a valuable tool for diagnosing various pathologies. Various methods for SNP detection have been reported including real-time PCR performed with primers and a probe spanning the SNP site [25].

The aim of this study was to develop a new approach for the subgenotyping of human HAV based on six simplex SNP genotyping RT-qPCR assays and to apply this approach to human clinical samples.

## Results

### Design of HAV subtype RT-qPCR assays

**Figure 1:**
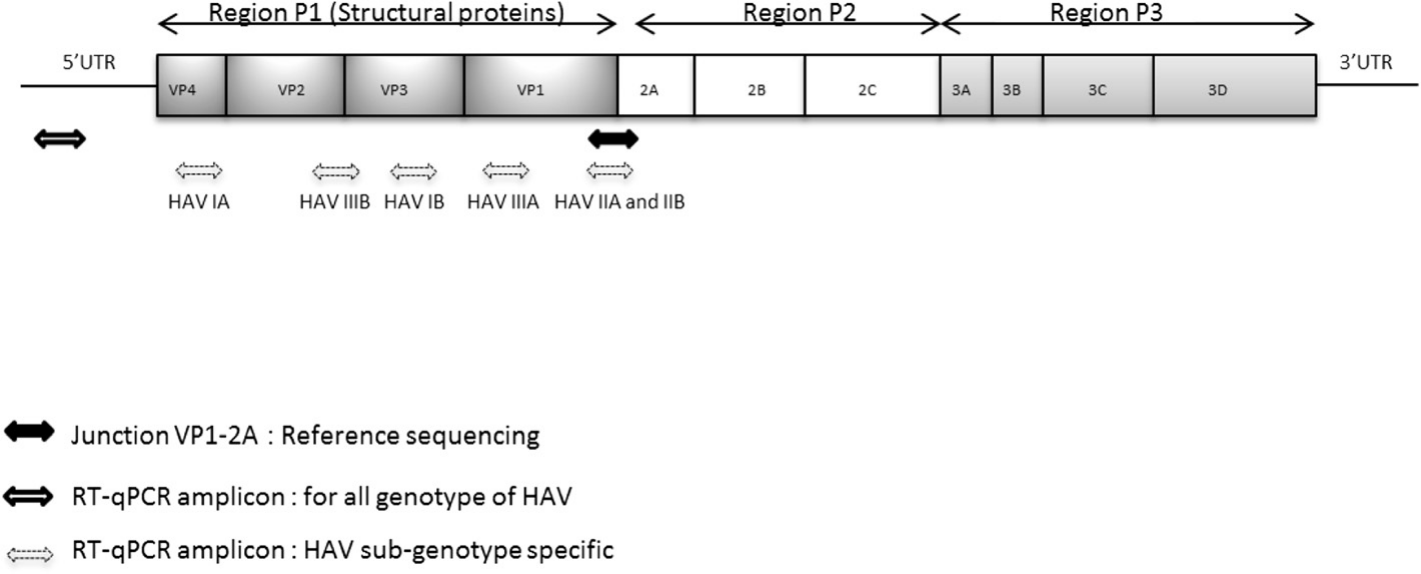
**HAV genome regions targeted for genotyping.** The different genomic regions used to identify each HAV genotype are represented below the HAV genome scheme.

The HAV subtype RT-qPCR assays were designed to give subtype-specific amplification on the basis of SNP differentiating the targeted subtype from the others. In other words, at the SNP position, the same nucleotide was found for all the subtypes except for the subtype of targeted HAV. Consequently, different regions of HAV genome were chosen given their subtype specificity and the absence of major nonspecific homologies on BLAST analysis. Moreover, degenerated bases were used to detect genetic variation within a given subtype (Table 1; Table 2; Figure 1).

**Table 1:** **GenBank accession numbers for HAV isolates**

**Genotype**	**Reference strain**	**GenBank accession number**
HAV IA	AB020564.1	EU526088; EU526089; EU131373; AB020567; X75216; EF406357; AB623053; X83302; X75214; AB020569; AB618531; AB020565; K02990; AB618529; AF485328; EF207320; HM769724; AB020564; EU251188; X75215; AB020568; HQ437707; AF512536; AB020566; AF357222
HAV IB	M14707	HQ246217; NC_001489; M14707; HV192265; FB746524; M59810; EF406361; EF406359; EF405360; DQ646426; EF406363; EF406362; AF268396; M59809; M16632; M59808; EF406358; AF314208; M20273
HAV IIA	AY644676.1	AY574059; AY644676; GU390574; GU390572; GU390577; GU390576
HAV IIB	AY644670.1	AY644670; Z77248; Z77247; Z77245; Z77244; Z77243; Z77246
HAV IIIA	AB279732.1	AB279732; FJ360735; EU011791; FJ360730; FJ360733; DQ991030; AB279734; DQ991029; FJ360732; FJ360734; AB279733; FJ360731
HAV IIIB	AB279735.1	AB279735; AB258387; AB425339; AB300205

Nucleic acid sequences were used to design primers and probes sets.

**Table 2:** **Sequences of primers and probes in the direction 5’-3’**

**Genotype**	**Reference strain**		**Sequence**	**Position**
HAV IA	AB020564.1	Forward primer	GCA TTT AGG TTT TTC CTC ATT	702-722
		Reverse primer	TCA ACK GAC TGA ATC ATT	837-820
		Probe	TCC AAA CAA GG**A** ATT TTC CAG AC	743-765
HAV IB	M14707	Forward primer	AAG CTT ATT GTG TAY TGT TAT	2070-2090
		Reverse primer	CAG AAT CAT CTC CAA CYT	2223-2208
		Probe	TTC TCC TTC TAA **C**GT TGC TTC CCA	2103-2125
HAV IIA	AY644676.1	Forward primer	ACY ATG ATG AGC AGA ATT G	2978-2996
		Reverse primer	GCA TAT TTT AAT CTC TGC TT	3129-3110
		Probe	AGA CCT GGA ATC **G**TC AGT GGA TGA	3004-3027
HAV IIB	AY644670.1	Forward primer	GGA GAT TTG AAA GTC ATA TTG	3047-3067
		Reverse primer	TTC CTG GGC ATA CTT TAG	3135-3118
		Probe	AGT CTT AAT TC**T** TTG TAT GGT TTTC	3078-3094
HAV IIIA	AB279732.1	Forward primer	TCC CTT GGA TTT GAC AAT	2605-2622
		Reverse primer	RGT ATT RAA CCT AAC AGC	2763-2747
		Probe	AAT TAT AAC TGG **G**GC TAC TGA TGT T	2623-2647
HAV IIIB	AB279735.1	Forward primer	AAT CCG ATG CTT CTC AAG	1560-1577
		Reverse primer	GCC TTC CTG AAT GGT ATT	1841-1824
		Probe	AAA ATT ACA CA**C** TTY ACA ACY TGG A	1589-1613
HAV 5’UTR	M14707	Forward primer*	TCA CCG CCG TTT GCC TAG	68-85
		Reverse primer*	GGA GAG CCC TGG AAG AAA G	241-223
		Probe*	CCT GAA CCT GCA GGA ATT AA	169-150

The specific genotype SNP is in bold. Probes are FAM-BHQ except HAV 5’UTR which is FAM-MGB. *: Costafreda et al. [19].

### Sensitivity of subtype-specific RT-qPCR assays

The sensitivity of the simplex subtype–specific RT-qPCR assays was evaluated with serial 10-fold dilutions of *in vitro* transcribed RNA for HAV IIA, IIB, IIIA and IIIB and genomic RNA for HAV IA and IB. From 5 × 10^5^ to 5 genome copies/assay for IA, IB, IIA, IIB, and IIIB and from 5 × 10^7^ to 5 × 10^2^ genome copies/assay for IIIA were tested. As shown on Table 3, mean RT-qPCR efficiency, derived from the slope parameters, ranged from 83.9% for IIIA to 109% for IB. R^2^ values were ≥0.898. The limit of detection (LOD) obtained for IA, IB, IIA and IIIB was 500 genome copies/assay, whereas the LOD of IIB was 50 genome copies/assay and the LOD of IIIA was 5000 genome copies/assay. The LOD of the consensus RT-qPCR assay [19] was in the same range as that of the subtyping RT-qPCR assays at 500 genome copies/assay.

**Table 3:** **Characteristics of RT-qPCR standard curves**

**Genome copies/RT-qPCR assay**	**Mean Ct values +/− SD**						
	**HAV IA**	**HAV IB**	**HAV IIA**	**HAV IIB**	**HAV IIIA**	**HAV IIIB**	**HAV 5'UTR**
5 x 10^7^	/	/	/	/	26,56 ± 0,89	/	/
5 x 10^6^	/	/	/	/	29,49 ± 0,88	/	/
5 x 10^5^	26.28 ± 0.59	26.54 ± 0.95	26.64 ± 0.60	22.25 ± 0.45	32.97 ± 0.94	24.50 ± 0.50	27.07 ± 1.00
5 x 10^4^	30.01 ± 0.58	29.60 ± 0115	30.26 ± 0.52	25.53 ± 0.35	36.90 ± 1.69	27.65 ± 0.48	30.76 ± 0.74
5 x 10^3^	33.34 ± 0.66	32.66 ± 1.47	33.82 ± 0.56	28.62 ± 0.66	**41.34 ± 0.69**	31.58 ± 0.47	33.32 ± 0.79
5 x 10^2^	**36.28 ± 0.69**	**36.16 ± 1.48**	**36.59 ± 0.79**	32.00 ± 0.67	nd	**35.64 ± 1.48**	**37.16 ± 1.83**
5 x 10^1^	nd	nd	nd	**35.79 ± 1.09**	/	nd	nd
5 x 10^0^	nd	nd	nd	nd	/	nd	nd
E	99.0%	109.0%	95.8%	100.8%	83.9%	86.5%	102.2%
R^2^	0.974	0.898	0.973	0.982	0.952	0.966	0.916

Parameters of RT-qPCR amplification curves obtained for HAV detection by the RT-qPCR reference method and HAV subgenotyping by RT-qPCR assays. The limit of detection (LOD) has been defined as the lowest amount of HAV detected in the three experiments and is shown in bold. nd: not detected. / : not analyzed.

### Specificity of subtype-specific RT-qPCR assays

The specificity of the RT-qPCR assays was assessed by testing HAV RNA of each subtype at a concentration of 5 × 10^4^ genome copies/assay with all the subtype-specific RT-qPCR assays. As shown on Table 4, detection of the specific target was observed for all assays with Ct values comprised between 25 and 37, consistent with assay sensitivity. All but one were entirely specific. The IIA-specific assay occasionally allowed amplification of the IIB target. However, this non-specific IIB amplification was not observed when as much as 5 × 10^4^ genome copies/assay of IIB RNA was tested in the presence of a low concentration of the specific IIA target (50 genome copies/assay) (data not shown).

**Table 4:** **Specificity of subgenotyping RT-qPCR assays**

	**Set primers/probe**						
		**HAV IA**	**HAV IB**	**HAV IIA**	**HAV IIB**	**HAV IIIA**	**HAV IIIB**
Sample	HAV IA	30.01 ± 0.58 (6/6)	-	-	-	-	-
	HAV IB	-	29.60 ± 1.15 (6/6)	-	-	-	-
	HAV IIA	-	-	30.57 ± 0.87 (6/6)	-	-	-
	HAV IIB	-	-	35.02 ± 1.31 (4/6)	25.53 ± 0.35 (6/6)	-	-
	HAV IIIA	-	-	-	-	36.90 ± 1.69 (6/6)	-
	HAV IIIB	-	-	-	-	-	27.65 ± 0.48 (6/6)

Six subtyping RT-qPCR assays were tested with 5 x 10^4^ genome copies/assay for all subtypes of HAV in duplicate in three different experiments. Results are expressed as means cycle threshold (Ct) values ± standard deviations (SD). The number of positive Ct values is given in parentheses.

### Fecal and serum samples analysis

Human clinical fecal and serum samples were genotyped by sequencing the VP1/2A region, as described [26] and provided by the NRC. Then, they were tested with the consensus RT-qPCR assay [19] and with all the subtype-specific RT-qPCR assays separately (Tables 5 and 6).

**Table 5:** **Stool samples analysis**

**Stool**	**Age/Sex**	**Transaminases IU/mL**	**Travel**	**Genotype by sequencing**	**HAV genome copies/g of stool**							**Difference of quantification between 5’UTR and subtype RT-qPCR assays (log**_**10**_ **)**	**Difference of quantification between IA and IIA subtype RT-qPCR assays (log**_**10**_ **)**
					**5’UTR**	**IA**	**IB**	**IIA**	**IIB**	**IIIA**	**IIIB**		
0780627147	21-30/M	NC	none	IA	2.30 x10^7^	1.93 x10^8^	-	-	-	-	-	IA : −0.92	-
1280210015	51-60/F	5680	Senegal	IB	1.50 x10^10^	-	7.19 x10^10^	-	-	-	-	IB : −0.68	-
1280514230	51-60/F	7191	Benin	IB	6.85 x10^10^	-	6.37 x10^10^	-	-	-	-	IB : +0.03	-
1181216151	51-60/F	3623	none	IA	7.75 x10^11^	5.75 x10^11^	-	3.85 x10^6^	-	-	-	IA : +0.13 ; IIA : +5.30	−5.17
078014121	51-60/F	NC	Morocco	IB	1.12 x10^7^	-	3.73 x10^5^	-	-	-	-	IB : +1.48	-

Each sample was tested with the reference RT-qPCR assay targeting the 5’UTR of HAV and the 6 genotype-specific RT-qPCR assays. The subtyping results were compared with those obtained with sequencing by the NRC. Concentrations are given in genome copies per gram of stool. NC = Not communicated. The difference of quantification between 5’UTR and subtype RT-qPCR assays is calculated by the formula: (log_10_ (genomes copies determined by reference RT-qPCR/genomes copies determined by subgenotyping RT-qPCR assays)). The difference of quantification between IA and IIA subtypes by RT-qPCR assays is calculated by the formula: (log_10_ (genomes copies determined by IA RT-qPCR/genomes copies determined by IIA RT-qPCR assays)).

**Table 6:** **Serum samples analysis**

**Serum**	**Age/Sex**	**Transaminases IU/mL **	**Travel**	**Genotype by sequencing**	**HAV genome copies/μl of serum**							**Difference of quantification between 5’UTR and subtype assays (log**_**10**_ **)**	**Difference of quantification between IA and IB subtype assays (log**_**10**_ **)**
					**5’UTR**	**IA**	**IB**	**IIA**	**IIB**	**IIIA**	**IIIB**		
1310074855	61-70/F	3935	Ethiopia	IB	1.50 x10^8^	-	2.87 x10^8^	-	-	-	-	IB : −0.28	-
1310016965	11-20/M	400	NC	IB	3.13 x10^5^	-	4.18 x10^3^	-	-	-	-	IB : +1.87	-
1311012387	1-10/F	NC	NC	IA	6.37 x10^4^	9.26 x10^4^	-	-	-	-	-	IA : −0.16	-
1311018436	1-10/M	NC	NC	IA	2.86 x10^4^	3.60 x10^4^	-	-	-	-	-	IA : −0.10	-
1311072234	31-40/F	2018	NC	IA	7.36 x10^4^	7.31 x10^4^	8.58 x10^3^	-	-	-	-	IA : 0 ; IB : +0.93	−0.93
1310064958	61-70/M	2314	Guadeloupe	IA	4.73 x10^5^	5.50 x10^5^	-	-	-	-	-	IA : −0.07	-
1311009716	51-60/M	2339	NC	IB	1.70 x10^6^	-	2.30 x10^6^	-	-	-	-	IB : −0.13	-
1311071503	1-10/M	358	NC	IA	2.72 x10^5^	2.85 x10^6^	3.22 x10^4^	-	-	-	-	IA : −1.02 ; IB : +0.93	−1.95
1310024892	1-10/M	NC	Morocco	IA	6.14 x10^6^	6.47 x10^6^	-	-	-	-	-	IA : −0.02	-
1310066012	1-10/F	1113	Morocco	IA	2.04 x10^3^	2.48 x10^3^	-	-	-	-	-	IA : −0.09	-
1309036458	11-20/M	3393	Cameroun	IIA	3.42 x10^5^	-	-	4.66 x10^6^	-	-	-	IIA : −1.13	-
1309064503	1-10/F	2470	Algeria	IA	3.27 x10^3^	3.99 x10^3^	-	-	-	-	-	IA : −0.09	-
1309044888	11-20/M	1352	Guinea	IB	4.10 x10^5^	-	7.00 x10^5^	-	-	-	-	IB : −0.23	-
1310077770	31-40/M	NC	Ethiopia	IB	1.48 x10^6^	-	4.35 x10^6^	-	-	-	-	IB : −0.47	-
1310044717	51-60/F	6500	NC	IB	7.16 x10^5^	-	1.32 x10^6^	-	-	-	-	IB : −0.26	-
1311011402	1-10/M	993	Ethiopia	IB	7.89 x10^5^	-	1.38 x10^6^	-	-	-	-	IB : −0.24	-
1311018712	41-50/M	NC	Ethiopia	IB	4.63 x10^3^	-	1.22 x10^4^	-	-	-	-	IB : −0.42	-
1310005428	21-30/M	3400	Morocco	IA	5.87 x10^3^	1.52 x10^4^	1.37 x10^2^	-	-	-	-	IA : −0.41 ; IB : +1.63	−2.05
1311011353	41-50/F	1736	Ethiopie	IB	4.43 x10^4^	-	9.28 x10^4^	-	-	-	-	IB : −0.32	-
1310023611	21-30/F	1388	Tunisia	IA	3.84 x10^4^	2.09 x10^4^	4.57 x10^3^	-	-	-	-	IA : +0.26 ; IB : +0.92	−0.66
1380219001	51-60/M	4000	Madagascar	IIIA	4.73 x10^5^	-	-	-	-	3.10 x10^4^	-	IIIA : +1.18	-
1311018504	61-70/F	1334	NC	IB	6.96 x10^4^	-	2.09 x10^5^	-	-	-	-	IB : −0.48	-
1309047363	11-20/F	2000	NC	IB	9.76 x10^4^	-	1.30 x10^5^	-	-	-	-	IB : −0.12	-
1310011213	1-10/M	1831	Algeria	IA	7.89 x10^4^	6.68 x10^4^	4.77 x10^3^	-	-	-	-	IA : +0.07 ; IB : +1.22	−1.15
1309064723	11-20/F	1494	Morocco	IA	3.22 x10^4^	2.76 x10^4^	-	-	-	-	-	IA : +0.07	-
1309039311	1-10/M	NC	Guinea	IB	1.25 x10^5^	-	5.35 x10^5^	-	-	-	-	IB : −0.63	-
1310053557	21-30/M	269	NC	IA	9.38 x10^1^	6.33 x10^2^	-	-	-	-	-	IA : −0.83	-
1310078280	1-10/F	857	Tunisia	IA	6.06 x10^4^	3.41 x10^4^	-	-	-	-	-	IA : +0.25	-
1380311211	61-70/M	1269	Madagascar	IIIA	1.48 x10^4^	-	-	-	-	4.25 x10^4^		IIIA : −0.46	-
1311062298	11-20/F	940	Morocco	IB	2.95 x10^2^	2.64 x10^2^	-	-	-	-	-	IA : +0.05	-

Each sample was tested with the reference RT-qPCR assay targeting the 5’UTR of HAV and the 6 genotype-specific assays. The subtyping results were compared with those obtained with sequencing by the NRC. Concentrations are given in genome copies per μl of serum. NC = Not communicated. The difference of quantification between 5’UTR and subtype RT-qPCR assays is calculated by the formula: (log_10_ (genomes copies determined by reference RT-qPCR/genomes copies determined by subgenotyping RT-qPCR assays)). The difference of quantification between IA and IB subtypes by RT-qPCR assays is calculated by the formula: (log_10_ (genomes copies determined by IA RT-qPCR/genomes copies determined by IB RT-qPCR assays)).

Four of the five stool samples and 24 out of the 30 sera were detected by a single subtype-specific assay that provided a subtype result consistent with VP1/2A sequencing. The consensus and specific RT-qPCR assays gave similar results with differences of quantification that did not exceed 1.9 log_10_ (genome copies/μL or genome copies/g).

In stool sample 1181216151 provided as a IA subtype by the NRC, subtype-specific assays detected both IA and IIA RNA, with a IIA concentration 5.2 log_10_ lower than that of the IA subtype (Table 5). Similarly, in the 5 sera provided as the IA subtype by the NRC, subtype-specific assays detected both IA and IB RNA, with IB concentrations 0.7 to 2 log_10_ lower than IA. A single discrepant result was observed for serum sample 1311062298 provided as a IB subtype by VP1/ 2A region sequencing and identified as a IA subtype by the subtype-specific RT-qPCR assays (Table 6).

In conclusion, the subgenotyping RT-qPCR assays allowed detecting 100% (35/35) of the clinical samples for the presence of HAV. In total, 80.0% (28 samples) of the clinical samples were found to correlate with the genotyping by sequencing the VP1/2A region. Furthermore, positivity for more than one genotype identified by sequencing appeared in 17.1% (6 samples) of the clinical samples and a subtype discrepancy in 2.9% (1 sample) of the clinical samples.

## Discussion

Although HAV has been shown to possess a single conserved antigenic neutralization site [27] leading to a single serotype, HAV strains isolated from different parts of the world have been classified into six genotypes (I to VI), of which genotype I, II, and III can infect humans. Genotype I is the most prevalent worldwide, and subtype IA is more common than IB. The other human genotypes are infrequent. In areas of low endemicity such as the United States and Western Europe, IA dominates but all genotypes and subtypes have been reported [23,28,29]. Genotype II isolates were originally identified in France in 1979 and Sierra Leone in 1988 [1] and appear to be limited to West Africa [30]. Genotype III has been reported in many parts of the world [28] but is prevalent in the Indian subcontinent. An increase in genotype IIIA infections has been reported in Korea, Russia, Estonia and in Japan. Moreover, IIIA and IIIB co-circulate broadly with IA and IB strains [5].

Phylogenetic analysis is useful to trace back the geographical origin of a given strain and for tracking transmissions of HAV. Accurate typing of HAV from food samples could thus be helpful for transmission investigations. However, HAV typing from food samples by a classical sequencing approach is often impaired by the low contamination levels, and does not give access to potential contamination by several strains. Indeed, implicated items (such as seafood, fruits and salads) in foodborne outbreaks can harbour a heterogeneous HAV population that reflects the diversity of the viral strains circulating at the geographic location of item contamination [31].

Two commercial quantitative HAV RT-qPCR assays have been described. The detection limit was 2 TCID_50_ /mL for the Roche kit and 5 TCID_50_/mL for the Artus kit. Both kits have been found suitable for detection and quantification of HAV but only the Roche kit allowed the differentiation between genotype IA and IB after melting curve analysis [32]. The present study introduces six RT-qPCR-based assays for specific molecular genotyping of hepatitis A virus. To our knowledge, this is the first time that HAV subtyping has been achieved by specific qPCR probes. This subtype identification method can be implemented in diagnostic and research laboratories, avoiding post-PCR analysis and avoiding the problem of low viral loads in food samples.

All subtype assays were found suitable for quantification measurement for comparison with the data obtained with the reference RT-qPCR assay (detecting all genotypes). The minimal variations (around 1log_10_) observed for the quantification were potentially due to the differences in amplification efficiencies and calibration curves used. Most of the samples were correctly identified with regard to the genotype provided by VP1/2A sequencing. In 6 samples (1 stool and 5 sera), the specific RT-qPCR assay identified a major IA strain, the same one determined by VP1/2A sequencing, also in addition to a second subtype, present in a lower concentration.

The conventional genotyping used as a reference assay is a “golden standard assay”. The design of HAV subgenotyping RT-qPCR assays was based on SNP in the probe associated with degenerated bases in the primers to enhance the specificity. Nevertheless, cross-reactivity could be only definitively excluded with the entire genome sequencing for the tested samples. However, co-circulation of the subgenotypes IA and IIIA has been reported in India [33] and of IA, IB and IIIA in Korea [34]. Co-circulation of the subgenotypes IA and IB in South Africa, South America, Europe and the US and the existence of recombination events between subgenotypes have also been observed [35-37]. Indeed, HAV exploits all known mechanisms of genetic variation to ensure its survival, including mutation and recombination [38,39]. HAV recombination was originally reported in cell culture [40]. Its extent in nature was appreciated only recently [35,36,38,39,41] and it appears that recombination occurs along the entire length of the genome [38].

The present finding from the stool sample of a patient who had not traveled abroad may be due to a co-infection by IA and IIA subtypes. Indeed, HAV IA is the dominant strain in France but IIA strains have been isolated among French travelers returning from Africa as well as from autochthonous cases [30]. A co-infection rather than an event of recombination is suggested because of the huge difference in the concentration of the subtypes. Regarding these two signals, although non-specific amplification due to a very high viral load cannot be excluded, it should be noted that no IIA amplification was detected from any of the 14 HAV IA serum samples.

The discovery of a major IA signal, combined with a 10- to 100-fold lower IB signal in 5 sera from patients having traveled abroad (at least for three of them) may suggest either an event of recombination or, more likely, a co-infection. For these samples, the genome copy numbers determined by the 5’-UTR assay was not the sum of those determined by subgenotyping RT-qPCR assays together which can be explained by the lack of accurate quantification or by cross reactivity. As conventional Sanger sequencing does not allow accurate identification of multiple species within a sample, the hypotheses could be investigated by cloning and sequencing or by next generation sequencing.

A single sample from a patient contaminated in Morocco provided a discrepant result by specific RT-qPCR and sequencing; this sample may correspond to an IA/IB recombinant in the P1 region of the HAV genome since IA-specific amplification targets the VP4 region (nt 702 to 820) and sequencing targets the VP1/2A region (nt 2870 to 3381). The sequencing of this sample was attempted but has been unsuccessful may be because of the low viral load.

## Conclusions

It was concluded that the RT-qPCR assays developed in this study are suitable tools for quantification of HAV and subtype identification. They need to be validated by testing a larger number of clinical, environmental and food samples. Conventional genotyping used as a reference assay is a “golden standard assay”, and the RT-qPCR assays described here could be recommended as an additional test to the conventional genotyping and for use in cases of failure of the conventional typing method. They may be particularly useful for accurately tracing HAV in samples with low-level contamination such as food matrices, but also can provide easy identification of a co-infection in human samples.

## Methods

### Viral isolates

The genotype IB HM175/18f strain, clone B (VR-1402) was obtained from the American Type Culture Collection (ATCC). This clone replicates rapidly and has cytopathic effects in cell culture [40]. HAV stock was produced by propagation in foetal rhesus monkey kidney (FRhK-4) cells (ATCC, CRL-1688) [42] and titrated by plaque assay [43]. Results were expressed in plaque-forming units/mL (PFU/mL) and HAV stock contained 10^7^ PFU/mL. Aliquots of 100 μL were kept frozen at −80°C for later use.

### Clinical samples

Ethics statement: Hepatitis A virus infection is a notifiable disease in France. The current system of mandatory reporting was approved by the Commission Nationale de l’Informatique et des Libertés (deliberation n° 02–082, November 19 2002). Patients receive oral and written information on the finality of the notification and on the modalities of information recording. This information is available on line on the web site of the Institut de Veille Sanitaire (IVS) at http://www.invs.sante.fr/content/download/6498/42945/version/2/file/fiche_info_patient.pdf.

All clinical and biological parameters are treated anonymously. The virological surveillance of strain diversity is performed on stored samples obtained for hepatitis A diagnosis (no need for any additional blood draw). Diagnostic laboratories are asked to contribute to HAV strains surveillance by sending samples to the National Reference Centre (NRC) for HAV. All data and samples are anonymously collected and analyzed. The study was conducted in accordance with the ethics principles of the Declaration of Helsinki.

HAV genotyping from stools and serum samples collected by the French NRC for Hepatitis A was determined by sequencing of the VP1/2A junction region as previously described [26]. Stool samples were suspended in 10 mM Phosphate Buffered Saline (PBS), pH 7.4, to obtain a final 10% suspension (w/v), vortexed and centrifuged at 3000 g for 30 min at 4°C. Aliquots of 100 μL supernatant were kept frozen at −80°C for later use. Serum samples were kept frozen at −80°C until later use.

### Viral RNA extraction

Aliquots of frozen fecal samples or viral stocks were supplemented with NucliSens® easyMAG™ lysis buffer (BioMérieux, Marcy l’Etoile, France) up to 3 mL and subjected to the NucliSens® easyMAG™ platform (Biomérieux) for total nucleic acid extraction by the “off board Specific A protocol” according to manufacturer’s instructions. Nucleic acids were finally eluted in 70 μL of elution buffer and stored at −80°C.

Two hundred μL of frozen sera samples were subjected to the NucliSens® easyMAG™ platform (Biomérieux) for total nucleic acid extraction by the “Specific B protocol” according to manufacturer’s instructions. Nucleic acids were then eluted in 50 μL of elution buffer and stored at −80°C.

### HAV RNA *in vitro* transcripts

The cDNA corresponding to nucleotides 39–518 (5’UTR) of the IB genomic sequence (M59808.1) was cloned into the pGEM-T Easy vector (Promega, Charbonnières-les-Bains, France) and propagated in *E. coli* One Shot® TOP10F’ (Life technologies, Saint Aubin, France). High quality DNA plasmid containing HAV regions (p-HAV5) was purified using the Qiagen Plasmid midi kit (Qiagen, Courtaboeuf, France) according to the manufacturer’s protocol.

HAV cDNA of genotypes IIA, IIB, IIIA or IIIB corresponding respectively to the 588–3183, 587–3183, 618–3210 and 618–3210 positions of the genomic sequence (AY644676.1, AY644670.1, AB279732.1, AB279735.1) were cloned into the pBluescriptIISK + vector by Genecust (Dudelange, Luxembourg). All recombinant plasmids were purified by Genecust and used to produce RNA transcripts. HAV IIA, IIB, and IIIB DNA plasmids (0,5 μg) were digested with *Hind*III (Life technologies) and HAV 5’UTR and HAV IIIA DNA plasmids were digested with *Spe*I (Life technologies). Digested plasmids were transcribed by using the MEGAscript® kit (Life technologies) according to the manufacturer’s protocol. Synthesized RNA was treated twice with Turbo™ DNase (Life technologies) according to the manufacturer’s protocol in order to remove the DNA template following transcription, and purified by using the MEGAclear kit (Life technologies) according to manufacturer’s instructions. The synthesized RNA was confirmed with RT-qPCR and quantified by measuring absorbance at 260/280 nm with a Nanodrop ND-100 (Thermoscientific, France) and the free software available on the “http://endmemo.com/bio/dnacopynum.php” website. RNA stocks were diluted to contain 10^9^ copies/μL and aliquoted and stored at - 80°C.

Titers of the clarified fecal suspensions, serum samples and HM175/18f supernatants were obtained by RT-qPCR targeting the 5’UTR (see below), using a standard curve derived from ten-fold dilutions of the 5’UTR transcript RNA from p-HAV5. Titer was expressed in genome copies.

### Primers and probes

The RT-qPCR assay targeting the 5’UTR described by Costafreda et al. [19] was used to detect all HAV genotypes. This RT-qPCR assay is referred to as “consensus RT-qPCR”. Primers and probe sets were designed by using Beacon Designer software (Bio-Rad, Marnes-la-Coquette, France) to give subtype-specific amplification on the basis of single nucleotide polymorphisms. To identify genotype-specific conserved regions of HAV, complete sequences available from GenBank (NCBI) were aligned (Table 1) with MUSCLE software [44] and multiple alignment was visualized with JALVIEW software (version 2.8) [45]. Hydrolysis probes were labeled at the 5’ end with 6-carboxyfluorescein (FAM) and at the 3’ end with black hole quencher 1 (BHQ1) (Table 2 and Figure 1).

Primers and probes were purchased from Life Technologies or Eurofins MWG Operon (Les Ulis, France).

### RT-qPCR conditions

Quantitative one-step RT-PCR for detection of HAV was carried out on a CFX96™ real-time PCR detection system from Bio-Rad. Reactions were performed in a 15 μL reaction mixture containing 1X of RNA UltraSense™ master mix and 0.63 μL of RNA Ultrasense™ enzyme mix, which are components of RNA UltraSense™ One-Step Quantitative RT-PCR System (Life technologies), 2 U RNAse inhibitor (Life technologies), 5 μg of bovine serum albumin (Life Technologies), 500 nM of forward primer, 900 nM of reverse primer, 250 nM of probe, and 5 μL of sample. A negative control containing all the reagents except the RNA template was included in each set of reactions. The one-step RT-qPCR program involved 60 min reverse transcription of RNA at 55°C, followed by a 5 min denaturation step at 95°C, and finally 45 cycles of 15 s at 95°C, 1 min at 56°C and 1 min at 65°C. Fluorescence was automatically recorded by the instrument at the end of the elongation steps (1 minute at 65°C) for each amplification cycle. All samples were characterized by a corresponding cycle threshold (Ct) value. Negative samples gave no Ct value. For each specific RT-qPCR assay, a standard curve was generated using 10-fold dilutions of titered RNA corresponding to each subtype. For the consensus RT-qPCR assay, a standard curve was generated using 10-fold dilutions of titered RNA transcripts from p-HAV5. The slopes (S) of the regression lines were used to calculate the amplification efficiency (E) of the RT-qPCR reactions, according to the formula E =10|-1/s| -1 [46].

### Assay performance assessment

Genotype IB HAV RNA obtained from HM175/18f, genotype IA RNA obtained from a fecal sample (stool number 128061099) and genotype IIA, IIIA, IIB, IIIB RNA transcripts were used to determine the sensitivity and the specificity of the subgenotyping RT-qPCR assays. All samples were analyzed in duplicate in three different experiments resulting from 6 Ct values.

## Abbreviations

ATCC: American type culture collection
BLAST: Basic local alignment search tool
CEN: European committee for standardisation
cDNA: Complementary DNA
DNA: Deoxyribonucleic acid
HAV: Hepatitis A virus
ISO/TS: International organization for standardization/technical specification
LOD: Limit of detection
ORF: Open reading frame
NCBI: National center for biotechnology information
NRC: National reference centre
PBS: Phosphate-buffered saline
PFU: Plaque-forming units
RNA: Ribonucleic acid
RT-qPCR: Quantitative reverse transcriptase PCR
SNP: Single nucleotide polymorphism
UTR: Untranslated region

## References

[1] Robertson BH, Jansen RW, Khanna B, Totsuka A, Nainan OV, Siegl G, Widell A, Margolis HS, Isomura S, Ito K (1992). Genetic relatedness of hepatitis A virus strains recovered from different geographical regions. J Gen Virol.

[2] Lu L, Ching KZ, de Paula VS, Nakano T, Siegl G, Weitz M, Robertson BH (2004). Characterization of the complete genomic sequence of genotype II hepatitis A virus (CF53/Berne isolate). J Gen Virol.

[3] Costa-Mattioli M, Cristina J, Romero H, Perez-Bercoff R, Casane D, Colina R, Garcia L, Vega I, Glikman G, Romanowsky V, Castello A, Nicand E, Bassin M, Billaudel S, Ferre V (2002). Molecular evolution of hepatitis A virus: a new classification based on the complete VP1 protein. J Virol.

[4] Koopmans M, Duizer E (2004). Foodborne viruses: an emerging problem. Int J Food Microbiol.

[5] Vaughan G, Goncalves Rossi LM, Forbi JC, de Paula VS, Purdy MA, Xia G, Khudyakov YE (2014). Hepatitis A virus: host interactions, molecular epidemiology and evolution. Infect Genet Evol.

[6] Franco E, Meleleo C, Serino L, Sorbara D, Zaratti L (2012). Hepatitis A: epidemiology and prevention in developing countries. World J Hepatol.

[7] World Health Organization (2000). Hepatitis A vaccines. Wkly Epidemiol Rec.

[8] Mohd Hanafiah K, Jacobsen KH, Wiersma ST (2011). Challenges to mapping the health risk of hepatitis A virus infection. Int J Health Geogr.

[9] Anonymous: **Hepatitis A outbreak in Australia [Internet].** National Travel Health Network and Centre; 2009 November 13 [cited 2010 March 25]. Available from:http://www.nathnac.org/pro/clinical_updates/hepatitisaoutbreakaustralia_131109healthprofessionals.htm.

[10] Beuchat LR (2006). Vectors and conditions for preharvest contamination of fruits and vegetables capable of causing enteric diseases. Brit Food J.

[11] Gallot C, Grout L, Roque-Afonso A-M, Couturier E, Carrillo-Santisteve P, Pouey J, Letort M-J, Hoppe S, Capdepon P, Saint-Martin S, De Valk H, Vaillant V (2011). Hepatitis A associated with semidried tomatoes, France, 2010. Emerg Infect Dis.

[12] Hernández F, Monge R, Jiménez C, Taylor L (1997). Rotavirus and hepatitis A virus in market lettuce (*Latuca sativa*) in Costa Rica. Int J Food Microbiol.

[13] Lacey S, Tracy L, Hammond R, Revill P, Donnan E, Lalor K, Bowden S (2009). Hepatitis A Virus Outbreak: Molecular Epidemiology Indicates a Common Source.

[14] Petrignani M, Verhoef L, van Hunen R, Swaan C, van Steenbergen J, Boxman I, Ober HJ, Vennema H, Koopmans M (2010). A possible foodborne outbreak of hepatitis A in the Netherlands, January–February 2010. Euro Surveill.

[15] Petrignani M, Harms M, Verhoef L, van Hunen R, Swaan C, van Steenbergen J, Boxman I, Perani Sala R, Ober H, Vennema H, Koopmans M, van Pelt W (2010). Update: a food-borne outbreak of hepatitis A in the Netherlands related to semi-dried tomatoes in oil, January–February 2010. Euro Surveill.

[16] Rosenblum LS, Mirkin IR, Allen DT, Safford S, Hadler SC (1990). A multifocal outbreak of hepatitis A traced to commercially distributed lettuce. Am J Public Health.

[17] Koopmans M, von Bonsdorff CH, Vinjé J, de Medici D, Monroe S (2002). Foodborne viruses. FEMS Microbiol Rev.

[18] Sánchez G, Bosch A, Pintó RM (2007). Hepatitis A virus detection in food: current and future prospects. Lett Appl Microbiol.

[19] Costafreda MI, Bosch A, Pinto RM (2006). Development, evaluation and standardization of a real time TaqMan reverse transcription-PCR assay for quantification of hepatitis A virus in clinical and shellfish samples. Appl Environ Microbiol.

[20] ISO/TS 15216–1 (2013). Microbiology of Food and Animal Feed -- Horizontal Method for Determination of Hepatitis A Virus and Norovirus in Food Using Real-Time RT-PCR -- Part 1: Method for Quantification.

[21] ISO/TS 15216–2 (2013). Microbiology of Food and Animal Feed -- Horizontal Method for Determination of Hepatitis A Virus and Norovirus in Food Using Real-Time RT-PCR -- Part 2: Method for Qualitative Detection.

[22] Lees D, CEN WG6 TAG4 (2010). International standardization of a method for detection of human pathogenic viruses in molluscan shellfish. Food Environ Virol.

[23] Nainan OV, Xia G, Vaughan G, Margolis HS (2006). Diagnosis of hepatitis a virus infection: a molecular approach. Clin Microbiol.

[24] Tjon G, Xiridou M, Coutinho R, Bruisten S (2007). Different transmission patterns of hepatitis A virus for two main risk groups as evidenced by molecular cluster analysis. J Med Virol.

[25] Woodward J (2014). Bi-allelic SNP genotyping using the TaqMan(®) assay. Methods Mol Biol.

[26] Schwarz NG, Revillion M, Roque-Afonso AM, Dussaix E, Giraud M, Liberpre C, Couturier E, Delarocque Astagneau E (2008). A food-borne outbreak of hepatitis A virus (HAV) infection in a secondary school in Upper Normandy, France, in November 2006. Euro Surveill.

[27] Stapleton JT, Lemon SM (1987). Neutralization escape mutants define a dominant immunogenic neutralization site on hepatitis A virus. J Virol.

[28] Costa-Mattioli M (2003). Genetic variability of hepatitis A virus. J Gen Virol.

[29] Tjon GM, Wijkmans CJ, Coutinho RA, Koek AG, van den Hoek JA, Leenders AC, Schneeberger PM, Bruisten SM (2005). Molecular epidemiology of hepatitis A in Noord-Brabant, The Netherlands. J Clin Virol.

[30] Desbois D, Couturier E, Mackiewicz V, Graube A, Letort MJ, Dussaix E, Roque-Afonso AM (2010). Epidemiology and genetic characterization of hepatitis A virus genotype IIA. J Clin Microbiol.

[31] Vaughan G, Xia G, Forbi JC, Purdy MA, Rossi LM, Spradling PR, Khudyakov YE (2013). Genetic relatedness among hepatitis A virus strains associated with food-borne outbreaks. PLoS One.

[32] Sánchez G, Populaire S, Butot S, Putallaz T, Joosten H (2006). Detection and differentiation of human hepatitis A strains by commercial quantitative real-time RT-PCR tests. J Virol Methods.

[33] Hussain Z, Das BC, Husain SA, Asim M, Chattopadhyay S, Malik A, Poovorawan Y, Theamboonlers A, Kar P (2005). Hepatitis A viral genotypes and clinical relevance: clinical and molecular characterization of hepatitis A virus isolates from northern India. Hepatol Res.

[34] Song HU, Hwang SG, Kwon CI, Lee JE, Ko KH, Hong SP, Park PW, Rim KS (2009). Molecular epidemiology of hepatitis A virus in the South-East area of Gyeonggi-do in Korea. Yonsei Me J.

[35] Aguirre S, Malirat V, Scodeller E, Mattion N (2011). First full-length genomic sequence of a hepatitis A virus isolated in Argentina shows recombination between subgenotypes IA and IB. Virus Res.

[36] Liu W, Zhai J, Liu J, Xie Y (2010). Identification of recombination between subgenotypes IA and IB of hepatitis A virus. Virus Genes.

[37] Villar LM, Morais LM, Aloise R, Melo MM, Calado IA, Lampe E, Gaspar AM (2006). Co-circulation of genotypes IA and IB of hepatitis A virus in Northeast Brazil. Braz J Med Biol Res.

[38] Belalov IS, Isaeva OV, Lukashev AN (2011). Recombination in hepatitis A virus: evidence for reproductive isolation of genotypes. J Gen Virol.

[39] Cristina J, Costa-Mattioli M (2007). Genetic variability and molecular evolution of hepatitis A virus. Virus Res.

[40] Lemon SM, Murphy PC, Shields PA, Ping LH, Feinstone SM, Cromeans T, Jansen RW (1991). Antigenic and genetic variation in cytopathic hepatitis A virus variants arising during persistent infection: evidence for genetic recombination. J Virol.

[41] Costa-Mattioli M, Ferré V, Casane D, Perez-Bercoff R, Coste-Burel M, Imbert-Marcille BM, Andre EC, Bressollette-Bodin C, Billaudel S, Cristina J (2003). Evidence of recombination in natural populations of hepatitis A virus. Virology.

[42] Cromeans T, Sobsey MD, Fields HA (1987). Development of a plaque assay for a cytopathic, rapidly replicating isolate of hepatitis A virus. J Med Virol.

[43] Dubois E, Hennechart C, Deboosère N, Merle G, Legeay O, Burger C, Le Calvé M, Lombard B, Ferré V, Traoré O (2006). Intra-laboratory validation of a concentration method adapted for the enumeration of infectious F-specific RNA coliphage, enterovirus, and hepatitis A virus from inoculated leaves of salad vegetables. Int J Food Microbiol.

[44] Edgar RC (2004). MUSCLE: multiple sequence alignment with high accuracy and high throughput. Nucleic Acids Res.

[45] Waterhouse AM, Procter JB, Martin DMA, Clamp M, Barton GJ (2009). Jalview version 2: a multiple sequence alignment and analysis workbench. Bioinformatics.

[46] Tichopad A, Dilger M, Schwarz G, Plaffl MW (2003). Standardized determination of real-time PCR efficiency from a single reaction set-up. Nucleic Acids Res.

